# Rare *Cryptosporidium hominis* Subtype Associated with Aquatic Center Use

**DOI:** 10.3201/eid1408.080115

**Published:** 2008-08

**Authors:** Corinne S.L. Ong, Simon Chow, Reka Gustafson, Candace Plohman, Robert Parker, Judith L. Isaac-Renton, Murray W. Fyfe

**Affiliations:** * University of British Columbia, Vancouver, British Columbia, Canada; † Fraser Health Authority, Surrey, British Columbia, Canada

**Keywords:** Cryptosporidium, subtype, recreational, letter

**To the Editor:** Cryptosporidiosis is the most frequently reported gastrointestinal illness in outbreaks associated with treated (disinfected) recreational water venues in the United States (*1*). In 2003, an increased number of cryptosporidiosis cases occurred in the Tri-Cities area of the Lower Mainland region (near Vancouver), in British Columbia, Canada. Although all cases were associated with the use of a community aquatic center, their onset dates were spread over a 3-month period, and the link between cases was unclear. The aim of this study was to determine if the cases in this disease cluster were related. Although suitable molecular markers had yet to be defined at the time of the outbreak, recent reports on the use of the gp60 gene for subtyping in molecular epidemiologic studies (*2*,*3*) have enabled us to reanalyze the isolates and report these results.

Fifteen laboratory-confirmed cases were identified from October 15 to December 5, 2003. This number was in excess of the anticipated incidence rate for this community, which averaged 5 reported cryptosporidiosis cases per year. During the period of investigation, an incident of fecal contamination at the aquatic center on October 10, 2003, was documented and remediation involved increasing the free chlorine concentration. Because the regional health authority was concerned about the increased number of cases, the facility closed voluntarily on December 5 for further remediation. However, recorded free chlorine concentrations did not exceed 2.0 ppm at any time during the investigative period (October 5–December 31).

The health authority released a public advisory encouraging those who used the facility to submit fecal specimens for laboratory testing. The health authority also sent letters to family physicians in the area, informing them of the disease cluster and requesting that unpreserved stool specimens be collected, in addition to the formalin-fixed specimens, for routine diagnostic testing. Nine fecal specimens were collected from clinically symptomatic case-patients with histories of exposure to the implicated aquatic center. Five specimens were selected by using the criteria that they were from patients with laboratory-confirmed cryptosporidiosis cases from 5 separate households. The specimens were then coded for anonymity before subsequent molecular analysis. Genomic DNA was extracted from purified *Cryptosporidium* oocysts by freeze-thawing, and the species was determined by PCR amplification and sequencing of the 18S rRNA gene as described previously (*4*). The gp60 gene was also amplified by PCR by using primers described by Ong and Isaac-Renton (*5*). DNA sequences of amplicons were determined by cycle sequencing and assembled as described previously (*4*,*5*). The gp60 allele and subtype were identified by multiple sequence alignment with GenBank reference sequences and phylogenetic analysis that used ClustalX version 1.8 (www.clustal.org) as well as manual quantification of microsatellite repeats.

The 18S rRNA and gp60 genes were amplified successfully from 4 specimens. On the basis of the 18S rRNA gene sequence, all case-patients were infected with *Cryptosporidium hominis*, a species associated primarily with human-to-human transmission. The gp60 sequences from all 4 case-patients were identical and were subtype IdA19, a rarely reported subtype of *C. hominis*. Globally, most reports of the gp60 Id allele, such as 9 reported cases from Australia, have identified the IdA15G1 subtype (*3*). Another subtype, IdA18, was isolated from 5 case-patients in a 1997 foodborne outbreak in Spokane, Washington (*6*). To date, the IdA19 subtype has been identified in only 1 sporadic case, in northern Ontario (*7*), and a subset of cases (seven 1dA19 and 2 mixed IdA19 and IbA10G2) in the 2001 waterborne outbreak in North Battleford, Saskatchewan (*5*,*8*). The IdA19 subtype is identical in sequence to the IdA18 subtype except for 1 extra TCA repeat in the microsatellite region. Neither subtype has been reported anywhere in the world except in Canada and the Pacific Northwest.

Because cases from all previous *C. hominis* outbreaks of cryptosporidiosis in British Columbia have been caused by the IbA10G2 subtype, the most prevalent subtype in sporadic and outbreak cases around the world (*2*,*5*,*9*,*10*), our results indicate the presence of a new subpopulation of *C. hominis* parasites that could cause future disease outbreaks. The identification of the same subtype in all 4 case-patients with cryptosporidiosis associated with the use of a community aquatic center was consistent with their exposure history and confirmed that all cases were linked epidemiologically. However, the association between the single northern Ontario sporadic case and the larger number of Saskatchewan and British Columbia outbreak cases is uncertain. The association with the IdA18 subtype in the Washington foodborne outbreak is also unknown. Further research is needed to determine the distribution and prevalence of gp60 subtypes in Canada as well as other parts of the world before we can more clearly understand the transmission of the IdA19 subtype.
